# Improved complete genome sequence of *Bdellovibrio bacteriovorus* 109J, a widely studied laboratory strain of predatory bacteria

**DOI:** 10.1128/mra.01296-23

**Published:** 2024-06-07

**Authors:** Yuki Hoshiko, Miki Okuno, Takeshi Yamamoto, Toshinari Maeda, Yoshitoshi Ogura

**Affiliations:** 1 Division of Microbiology, Department of Infectious Medicine, Kurume University School of Medicine, Kurume, Fukuoka, Japan; 2 Department of Biological Functions Engineering, Graduate School of Life Science and Systems Engineering, Kyushu Institute of Technology, Kitakyushu, Fukuoka, Japan; The University of Arizona, Tucson, Arizona, USA

**Keywords:** predatory bacteria, *Bdellovibrio bacteriovorus*, genome

## Abstract

The complete genome sequence of *Bdellovibrio bacteriovorus* 109J, a well-studied laboratory strain of predatory bacteria, first determined in 2014. Here we report an improved complete genome sequence of *B. bacteriovorus* 109J, incorporating 16 assembly and 87 nucleotide corrections. This revised genome will be helpful to studies on the predatory bacteria.

## ANNOUNCEMENT

**Fig 1:**
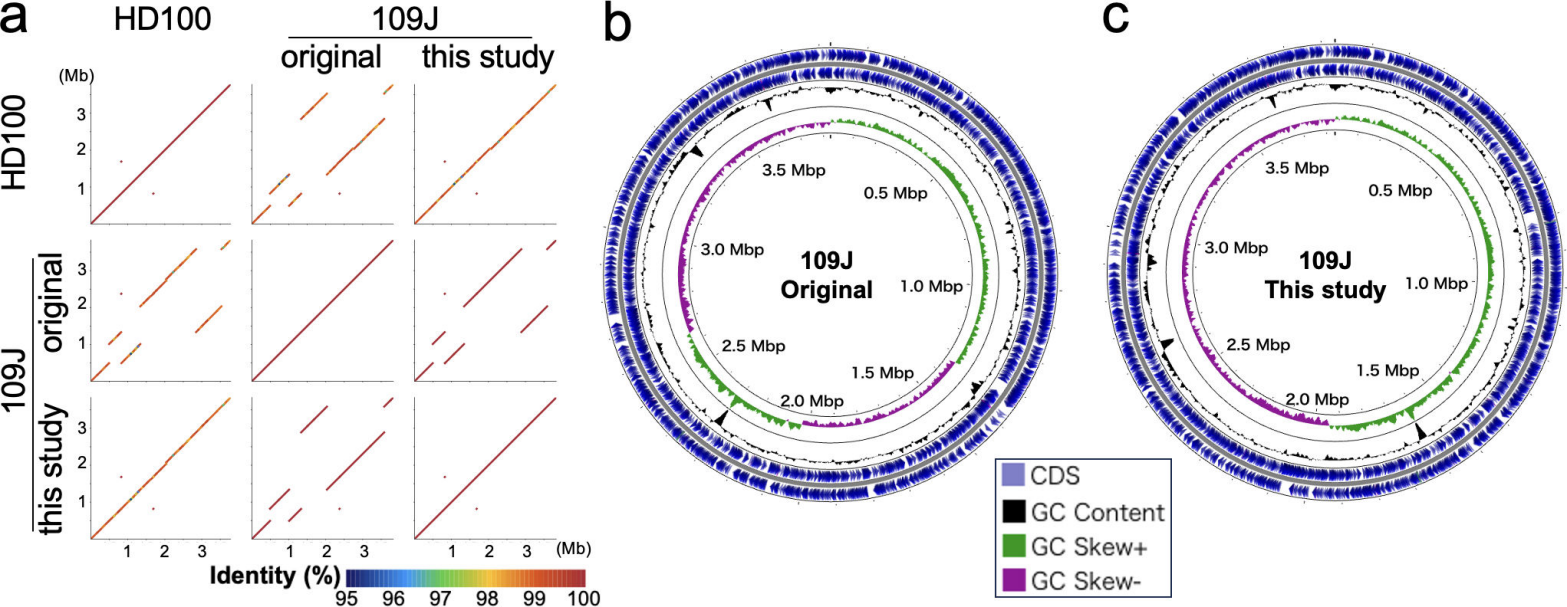
Dot plot comparison and circular map of *B. bacteriovorus* strains HD100 and 109J. (**a)** Dot plot matrix of the HD100, original 109J and improved 109J chromosomes. The start positions of their chromosome sequences were rearranged to the *dnaA* gene. The matrix was depicted by an in-house Python script based on the results of NCBI BLASTN search. (**b** and **c)**: Circular maps of the original (**b**) and improved (**c**) *B. bacteriovorus* 109J chromosome generated using Proksee server (https://proksee.ca/) (5) are shown.

*Bdellovibrio bacteriovorus*, a predatory Gram-negative bacterium, thrives by consuming the cellular contents of other Gram-negative bacteria as its source of nourishment (1). The laboratory strain 109J (ATCC 43826, US sewage isolate), in addition to the type strain HD100 (ATCC15356), is extensively employed in both basic and applied microbial studies (2, 3). The complete genome sequence of 109J was deposited in 2014 in NCBI database (4). Nonetheless, our dot-plot analysis of the 109J chromosome in comparison with the HD100 chromosome unveiled structural anomalies encompassing several large translocations therein (Fig. 1a). Furthermore, the 109J chromosome showed two unusual GC-skew shifts in addition to those at the replication origin and terminus regions (Fig. 1b). Another noteworthy consideration is that the deposited 109J genome was determined solely from low coverage (12×) of Illumina short-read data. Consequently, we performed the resequencing of the 109J genome, employing a combination of short- and long-read sequencing.

To extract 109J gDNA, a coculture of 109J, which has undergone eight-times passages from frozen stock, and *E. coli* BW25113, serving as host cells, was established by inoculation of 109J plaque (a 1.5 × 1.5 cm piece) and BW25113 overnight culture (10^9^ CFU/mL) into 50 mL of 50-fold diluted nutrient broth. Following a 48 h incubating at 37°C with shaking at 200 rpm, the coculture was filtered twice through a 0.45-µm pore size filter to remove *E. coli* cells. Subsequently, genomic DNA extraction from the filtrated 109J was performed using the DNeasy Blood & Tissue Kit (Qiagen). The library for short-read sequencing was prepared using the xGen DNA Library Prep EZ Kit (Integrated DNA Technologies) and NEBNext Multiplex Oligos for Illumina (96 Unique Dual Index Primer Pairs) (New England BioLabs) according to the manufacturer’s instructions. The library was subjected to sequencing on an Illumina NovaSeq X plus platform to generate 151 bp paired-end reads. For long-read sequencing, following cutoff of ~10 k-bp DNA fragments using the Short Read Eliminater XS kit (PacBio), the library was prepared using Rapid Barcoding Sequencing kit SQK-RBK004 (Oxford Nanopore Technologies). The library was loaded onto the R9.4.1 flow cell on a MinION Mk1C platform. The row data were base called with Guppy 6.5.7. Illumina reads were trimmed with Platanus_trim 1.1.0 (6) and Nanopore reads were filtered with NanoFilt 2.8.0 (7) to remove <5 kb reads. A hybrid assembly of both generated sequencing data was conducted by Unicycler v0.5.0 (8). Annotation was performed using Prokka 1.14.6 (9). Default parameters were used for all software unless otherwise specified.

The improved 109J genome sequence consists of a 3,836,928 bp chromosome, exhibiting an enlargement of 6,501 bp in contrast to the original sequence (Table 1). Sequence comparison using MUMmer3.23 (10) unveiled 16 structural differences and 87 nucleotide substitutions between the original and improved genomes (Supplementary data). The chromosomal synteny has become well conserved between the HD100 and improved 109J genomes. Furthermore, the GC skew pattern of the improved 109J chromosome has assumed a normative configuration (Fig. 1c). The improved 109J genome sequence will be helpful to studies of *B. bacteriovorus*.

**TABLE 1:** Summary of the comparison of the original and improved *B. bacteriovorus* 109J genomes

Sequence data	109J (original)	109J (this study)
Number of reads	ND^*a*^	6,473,258
Total read length	ND	2,801,481,736
N50 of the Nanopore reads	ND	11,223
Coverage	12×	730×
Scaffolds	1	1
Total length [bp]	3,830,427	3,836,928
Ambiguous code	2	0
N50	3,830,427	3,836,928
GC [%]	50.65	50.65
CheckM completeness [%]	97.82	99.05
CheckM contamination [%]	1.98	0.82
Genes	3,641	3,671
CDSs	3,589	3,628
rRNA operons	1	2
tRNAs	35	36
Accession number	CP007656	AP029059

^*a*^ ND: No data.

## Data Availability

The raw read sequences and assembled/annotated genome have been deposited in the DDBJ. The accession number for BioProject is PRJDB17218. The accession number for the assembled genome and the raw read data are AP029059 and DRX503239-40, respectively. Supplementary data can be accessed at the BioStudies database under accession number S-BSST1264.
